# Validity and reliability of a Nigerian-Yoruba version of the stroke-specific quality of life scale 2.0

**DOI:** 10.1186/s12955-017-0775-9

**Published:** 2017-10-19

**Authors:** Marufat Oluyemisi Odetunde, Aderonke Omobonike Akinpelu, Adesola Christiana Odole

**Affiliations:** 1 Department of Physiotherapy, Obafemi Awolowo University Teaching Hospital, Ile-Ife, Nigeria; 2 Department of Physiotherapy, College of Medicine, University of Ibadan, Ibadan, Nigeria

**Keywords:** Yoruba SS-QoL 2.0, Yoruba WHOQoL-BREF, Validity, Reliability

## Abstract

**Background:**

Psychometric evidence is necessary to establish scientific integrity and clinical usefulness of translations and cultural adaptations of the Stroke-Specific Quality of Life (SS-QoL) scale. However, the limited evidence on psychometrics of Yoruba version of SS-QoL 2.0 (SS-QoL(Y)) is a significant shortcoming. This study assessed the test-retest reliability, internal consistency, convergent, divergent, discriminant and known-group validity of the SS-QoL(Y).

**Methods:**

Yoruba version of the WHOQoL-BREF was used to test the convergent and divergent validity of the SS-QoL(Y) among 100 consenting stroke survivors. The WHOQoL-BREF and SS-QoL(Y) was administered randomly in order to eliminate bias. The test-retest reliability of the SS-QoL(Y) was carried out among 68 of the respondents within an interval of 7 days. All respondents were purposively recruited from selected secondary and tertiary health facilities in South-west Nigeria. Data were analysed using descriptive statistics of mean and standard deviation, and inferential statistics of Spearman correlation, Cronbach’s alpha, Intra-class Correlation Coefficient (ICC), Independent t-test and One-way ANOVA. Alpha level was set at *p* < 0.05.

**Result:**

The physical health, psychological health, social relationship and environment domains on WHOQoL-BREF with correlation coefficient that ranged from 0.214 to 0.360 showed significant correlation with similar domains on SS-QoL(Y). Dissimilar domains between the two scales had *r* values from 0.035 to 0.366. Discriminant validity of SS-QoL(Y) showed that items’ *r* value ranged from 0.711 to 0.920 with their hypothesized domains. The scale demonstrated moderate to strong test-retest reliability with Intra-class correlation coefficient (ICC) for the domains and overall scores (*r* = 0.47 to 0.81) and moderate to high internal consistency (Cronbach’s alpha =0.61 to 0.82) for domains scores. These correlations were also significant for the domains and overall scores (*p* < 0.05). There were no significant differences across different age groups or gender for the domains or overall scores of SS-QoL(Y).

**Conclusions:**

Discriminant and known-group validity, test-retest reliability and internal consistency of the Yoruba version of the Stroke Specific Quality of Life 2.0 are adequate while the convergent and divergent validity are low but acceptable. The SS-QoL(Y) is recommended for assessing health-related quality of life among Yoruba stroke survivors.

**Electronic supplementary material:**

The online version of this article (10.1186/s12955-017-0775-9) contains supplementary material, which is available to authorized users.

## Background

Stroke has a detrimental effect on both short-term and long-term Health-Related Quality of Life (HRQoL) [1]. Disability resulting from stroke is a strong determinant of HRQoL [2]. The impact of stroke on Quality of Life (QoL) is necessary for a comprehensive assessment of health and health-care, as it is helpful in evaluating the effects of different interventions on stroke prevention and treatment [1]. Patients’ perspective on the consequences of disease and the therapeutic benefits is considered important in the evaluation of health care. Patient-reported outcome measures such as QoL have therefore been used to supplement clinical decisions made from clinician-based outcome measures [3].

The Stroke-Specific Quality of Life Scale (SS-QoL) developed by Williams et al. (1999) [4] is one of the most comprehensive [3] and frequently used patient-reported stroke-specific outcome measures [5–7]. The domains of disease-specific HRQoL measures are of central concern in terms of treatment goals [8]. Therefore, disease-specific HRQoL measures have been found to be very promising for use in capturing the diverse concerns of stroke populations [4, 9]. The SS-QoL version 2.0 is a stroke outcome measure designed to capture all domains meaningful to stroke patients [4]. It is a measure derived from a series of focused interviews with ischemic stroke survivors to assess quality of life [4]. The SS-QoL 2.0 consists of 49 items in 12 domains of self-care, vision, language, mobility, work/productivity, upper-extremity function, thinking, personality, mood, family roles, social roles and energy. Items are rated on a 5-point Likert scale with higher scores indicating better function. Compared with common generic HRQoL measures, the SS-QoL has a broader coverage of functions typically affected by stroke [4]. The SS-QoL 2.0 has been shown to demonstrate adequate to excellent internal consistency (Cronbach’s α >0.73) across the 12 domains with most domains moderately correlated (r^2^ range = 0.3 to 0.5) with similar domains of established outcome measures such as Barthel Index and General Health Survey Short Form (SF-36) (Saladin, 2000) [10].

The SS-QoL 2.0 has been cross-culturally adapted to many languages including Spanish [11], German [12], Danish [5, 13], Greek [14], Brazilian [15], Chinese [16], Turkish [17], Kannada(India) [18], Persian [19], Malayalam (India) [20], Hindi (India) [21] and the Yoruba version (SS-QoL (Y)) [22]. Some of these translations have required psychometric evidence beyond being available in another language, while some do not. For example, convergent validity of the SS-QoL was carried out in Danish version [13] using the generic SF-36 survey and National Institute of Health Stroke Scale; while the Turkish version [17] used the SF-36 survey and Katz Index of Activities of Daily Living respectively.

Cross-cultural adaptation of SS-QoL 2.0 into Yoruba was carried out by Akinpelu et al. [22], and it was reported to have fulfilled the initial criteria for validity. From the study of Akinpelu et al., moderate to high correlation (*r* = 0.54 to 0.89) was reported for domain scores on adapted English version and SS-QoL (Y) with no significant difference between adapted English version and SS-QoL (Y) except in work/productivity domain.

Instrument measurement properties such as validity and reliability are required for confidence in interpretation of measurements [23]. The availability of psychometrically sound translated versions of standardized health measuring scales increase the certainty of accurate measure [24, 25]. Further evaluation of the psychometric properties of the SS-QoL (Y) on the target population is necessary in order to establish its scientific integrity and clinical usefulness [26, 27]. The availability of psychometrically sound SS-QoL 2.0 in an indigenous Nigerian language such as Yoruba may promote the use of the scale among Yoruba population in Nigeria and other places where the language is spoken. This may also ensure that Yoruba stroke survivors who are not literate in English are not excluded from measurement of health variables that are important and meaningful to them.

The Yoruba language is the indigenous language of the people of South-western Nigeria. Yoruba are a nationality, a black people, the majority of whom live in the South Western part of Nigeria in West Africa; speaking a common language, Yoruba, which belongs to the Kwa group of the Niger-Congo linguistic family and has dialects not less than 12 [28]. There are substantial indigenous Yoruba communities in the Republic of Benin, Togo and Sierra Leone, with large groups of Yoruba migrants living in the United States and the United Kingdom [28]. Likewise, Diaspora Yoruba communities include Brazil, Cuba, Puerto Rico, Trinidad and the rest of the Caribbean as well as Asia [28].

In order to further validate the SS-QoL (Y), this study assessed the test-retest reliability, internal consistency, convergent, divergent, discriminant and known group validity of the SS-QoL (Y). The convergent and divergent validity of the SS-QoL (Y) was assessed by comparing it with the Yoruba version of the World Health Organization’s quality of life scale-short form (WHOQoL-BREF). We hypothesised that similar and dissimilar domains of Yoruba versions of SS-QoL 2.0 and WHOQoL-BREF would correlate significantly.

## Methods

### Participants

This study was conducted at the outpatient Physiotherapy departments of selected secondary and tertiary health institutions in South-western States of Nigeria (Oyo, Osun, Ogun, Lagos, Ondo and Ekiti states) from January 2013 to June 2014. A total of 100 stroke survivors consented for the study (61 males and 39 females). Participants were stroke survivors with first attack or recurrent stroke with different levels of motor impairment, stroke duration and cognitive abilities that understood the items on the questionnaires and chose a response option for each of the items. This is in line with recommendations of Lin et al. [29] in order to increase the external validity and generalizability of the results of the study.

Inclusion criteria for the study included being an indigenous Yoruba person, having a diagnosis of ischemic or haemorrhagic stroke of at least 1 month [4, 15] and having the required cognitive ability to complete the questionnaires. Exclusion criteria were stroke survivors on hospital admission, having dysphasia that interfered with meaningful communication; co-morbidities that concurrently affect Health-Related QoL (for example, class III or IV heart failure, severe pre-existing musculoskeletal disease limiting physical function, metastatic cancer, active psychiatric disease or dementia, and diagnosis of HIV infection or AIDS. The Health Research Ethics committee of University of Ibadan/University College Hospital Ibadan gave approval for the study.

### Instruments

The Yoruba version of the SS-QoL 2.0 (Additional file 1) and Yoruba version of WHOQoL-BREF (Additional file 2) were used in this study.

1. **Yoruba version of the SS-QoL 2.0 (SS-QoL (Y)):** The original English version of the SS-QoL 2.0 was cross-culturally adapted into the Yoruba language and an initial investigation on its validity was conducted among Yoruba stroke-survivors in the south-western Nigeria [22]. Significant correlations were reported between the domain scores and overall score on the English Version and the corresponding domain scores (*r* ranged from 0.54 to 0.89) and overall score (*r* = 0.79) on Final version of the SS-QoL (Y) 2.0. The scale was reported to demonstrated evidence of adequate construct validity. This Yoruba version of SS-QoL 2.0 was further assessed for validity, and reliability in the present study.
2. **Yoruba version of the World Health Organization Quality of Life BREF (WHOQoL-BREF):** The WHOQOL-BREF consists of one item each from the 24 facets included in the WHOQoL-100 making up the four domains of physical health (PH), psychological health (PSH), social relationship (SoR) and environment (Env) as well as two items from the overall quality of life and the general health [30]. Domain scores are scaled in a positive direction with higher scores denoting higher quality of life. The mean score of items within each domain is used to calculate the domain score. The mean score is then multiplied by four to make the scores comparable to the score of WHOQoL-100 [30]. This gives the domains scores in the range 4–20, which is transformed again to 0–100 scale. WHOQoL-BREF is a well validated, cross-cultural tool for measuring quality of life of patients with chronic diseases. The scale was translated into Yoruba language [31] among other languages. The Yoruba version of WHOQoL-BREF like the English version consists of 24 items in 4 domains and 2 items from the overall quality of life and the general health. The scale has been validated among Yoruba-speaking stroke survivors with evidence of moderate to high and significant correlation between it and English version (*r* = 0.695–0.859; *p* < 0.001) [31].

### Procedure

Demographic and clinical data of participants were obtained using a proforma. Convergent and divergent validity of the SS-QoL (Y) was assessed using the Yoruba version of WHOQoL-BREF among 100 Yoruba stroke survivors. Both scales were administered through face to face interview with paper and pen at the clinic by a trained research assistant. Face to face interview has been reported to be the least tasking mode of administration of questionnaire which stimulates accurate responses with the interviewer in maximum control of question order [32]. Both scales were administered in random order. The Yoruba version of SS-QoL 2.0 was re-administered on 68 of the participants at the interval of 7 days to assess its test-retest reliability [1, 11, 18].

## Data analysis

Descriptive statistics of mean and standard deviation were used to analyze domains and overall scores on the SS-QoL (Y). Convergent, divergent and discriminant (i.e. correlations between each item and its hypothesized domain) validity of the SS-QoL (Y) were tested using the Spearman’s correlation method. Known group validity was tested by comparing domain scores by gender and age groups using independent t-test and One-way ANOVA respectively. Intra-class correlation (ICC) was used to determine the reliability (test-retest) and Cronbach’s alpha values were determined for internal consistency of the SS-QoL (Y). SPSS version 16.0 (Chicago IL SPSS Inc.) was used to analyze data. Level of significance was set at *p* < 0.05.

## Results

The mean age and time from stroke of the respondents was 55.27 ± 12.34 years and 20.81 ± 33.71 months respectively while the median time from stroke onset was 11 months. The socio-demographic and clinical characteristics of the respondents are presented in Table 1. Seventy-one percent of the respondents had ischaemic stroke, 59% had right hemiplegia/hemiparesis and 48% of the respondents have had stroke for 6 months or more. The mean overall score on the SS-QoL (Y) was 171.02 ± 30.28 (Table 2).

Domains with similar items on the SS-QoL (Y) and WHOQoL-BREF were compared. The mean domain scores on WHOQoL-BREF was highest on Env. domain, followed by PSH domain (Table 3). The correlation between Physical Health (PH) domain on Yoruba version of WHOQoL-BREF and mobility, work and energy domains of SS-QoL (Y) were 0.28, 0.35 and 0.23 respectively which are significant. Significant correlation was found between Psychological Health (PSH) domain on Yoruba version of WHOQoL-BREF and thinking domain as well as between PSH and mood domain of SS-QoL 2.0 with r values of 0.24 and 0.36 respectively; Social Relationship (SoR) domain of Yoruba version of WHOQoL-BREF correlated significantly with Social Role domain of SS-QoL (Y) with r value of 0.26; while Environment domain on Yoruba version of WHOQoL-BREF correlated significantly with family role and social role domains on SS-QoL (Y) with r values of 0.24 and 0.21 respectively (Table 4). Domains with dissimilar items on the SS-QoL (Y) and WHOQoL-BREF were also compared. Weak to fair correlations (*r* ranged from 0.035 to 0.366) were demonstrated between dissimilar domains of the two scales. However, significant correlation was found between some of the dissimilar domains (Table 4). Out of the 12 domains of SS-QoL 2.0 three are related to physical health domain of WHOQoL-BREF while nine are unrelated; two are related to the psychological heath domain while 10 are unrelated; one is related to social relationship domain while 11 are unrelated; two are related to environment domain while 10 are unrelated (Table 4). For the known-group validity of the SS-QoL (Y) by gender and age, Table 5 shows the result of the independent t-test comparison of domains and overall scores by gender. The result showed no significant gender difference in the domains and overall scores (*p* > 0.05). Table 6 shows the result of the One-way ANOVA comparison of domains and overall scores by age group. There were no significant differences in the mean domains and overall scores on SS-QoL (Y) across various age groups (*p* > 0.05). The mean domain scores increased steadily across the age groups below 50 years, 50–59 years and 60–69 years on self-care, language, family role, social role and energy domains and the overall score. A decline in mean scores was observed for the oldest age group (≥70 years) on self- care, mobility, upper extremity, family role and social role domains while steady increase in the mean overall score was observed across all age groups.

**Table 1:** Demographic and Clinical Characteristics of Yoruba Stroke Survivors

Variables		Frequency	Percentage
Gender	Male	61	61
	Female	39	39
Age group (years)	< 50	23	23
	50–59	30	30
	60–69	16	16
	≥ 70	11	11
Stroke type	Haemorrhagic	29	29
	Ischaemic	71	71
Age at onset of stroke	< 50	23	23
	50–59	40	40
	60–69	26	26
	≥ 70	11	11
Affected side	Left	41	41
	Right	59	59
Time from Stroke	**<** 3 months	16	16
	3-6 months	19	19
	>6 months	48	48
	Not specified	17	17

**Table 2:** Participants’ Scores on the Yoruba versions of Stroke Specific Quality of Life 2.0

Domains	Mean	Standard Deviation	Minimum	Maximum
SC	17.43	5.28	5.00	25.00
V	13.62	2.20	5.00	15.00
L	21.48	4.78	5.00	25.00
M	21.05	5.50	6.00	30.00
W	8.44	3.85	3.00	15.00
UE	16.49	5.69	5.00	25.00
T	10.89	3.76	2.00	15.00
P	10.21	3.66	3.00	15.00
MD	19.26	4.77	7.00	25.00
FR	10.21	3.86	3.00	15.00
SR	11.26	5.21	5.00	25.00
E	10.58	3.64	4.00	15.00
O	171.02	30.28	94.00	230.00

*SC* self-care; *V* vision, *L* language, *M* mobility, *W* work, *UE* upper extremity, *T* thinking, *P* personality, *MD* mood, *FR* family role, *SR* social role, *E* energy, *O* overall

**Table 3:** Participants’ Scores on the Yoruba Version of WHOQoL-BREF

Domains	Mean	Standard Deviation	Minimum	Maximum
Physical Health	20.12	4.70	11.00	35.00
Psychological Health	20.29	4.05	9.00	30.00
Social Relationship	9.23	2.85	3.00	15.00
Environment	27.89	5.29	17.00	40.00
General Health	6.22	1.80	2.00	10.00

**Table 4:** Spearman’s Correlation of Yoruba versions of SS-QoL 2.0 and WHOQoL- BREF

Domains	PH		PSH		SoR		Env	
	r	*p*	r	*p*	r	*p*	r	*p*
SC	0.285	0.004*	0.244	0.015*	0.263	0.008*	0.170	0.092
V	0.193	0.056	0.194	0.054	0.071	0.488	0.267	0.007*
L	0.362	0.001*	0.200	0.047*	0.078	0.443	0.147	0.146
**M**	**0.275**	0.006*	0.149	0.142	0.366	0.001*	0.180	0.075
**W**	**0.354**	0.001*	0.158	0.118	0.236	0.019*	0.049	0.633
**UE**	0.162	0.102	0.094	0.356	0.142	0.160	0.098	0.335
**T**	0.035	0.731	**0.240**	0.017*	0.149	0.141	0.214	0.034*
**P**	0.157	0.121	0.167	0.099	0.210	0.037*	0.083	0.412
**MD**	0.119	0.239	**0.360**	0.001*	0.250	0.012*	0.289	0.004*
**FR**	0.089	0.383	0.314	0.002*	0.179	0.076	**0.236**	0.019*
**SR**	0.162	0.109	0.257	0.010*	**0.256**	0.010*	**0.214**	0.033*
**E**	**0.234**	0.020*	0.049	0.633	0.207	0.040*	0.120	0.238
**O**	0.361	0.001*	0.316	0.001*	0.341	0.001*	0.259	0.010*

Bold figures indicates similar domains

*SC* Self Care, *V* Vision, *L* Language, *M* Mobility, *W* Work, *UE* Upper Extremity, *T* Thinking, *P* Personality, *MD* Mood, *FR* Family Role, *SR* Social Role, *E* Energy, *O* Overall, *PH* Physical Health, *PSH* Psychological Health, *SoR* Social Relationship, *Env* Environment

*indicates significant correlation (*p* < 0.05)

**Table 5:** Independent t-test comparison of domains and overall score of the Yoruba version of the Stroke-specific Quality of Life Scale by gender

Domains	Gender		t-cal	*p*-value
	Male x̄ ±SD(*n* = 61)	Female x̄ ±SD(*n* = 39)		
SC	17.30 ± 4.98	17.64 ± 5.09	−0.34	0.74
V	13.56 ± 2.31	13.72 ± 2.10	−0.35	0.73
L	21.21 ± 5.23	21.95 ± 3.65	−0.76	0.45
M	21.18 ± 5.61	21.38 ± 6.53	−0.16	0.87
W	8.75 ± 3.63	8.18 ± 3.90	0.74	0.46
UE	18.25 ± 5.52	17.21 ± 5.76	−0.83	0.41
T	11.18 ± 3.70	10.46 ± 4.15	0.90	0.37
P	10.16 ± 3.54	10.63 ± 3.63	0.63	0.53
MD	19.10 ± 5.12	19.95 ± 4.83	−0.82	0.41
FR	10.56 ± 10.08	9.74 ± 4.39	0.94	0.35
SR	12.61 ± 6.40	13.10 ± 6.54	−0.38	0.71
E	10.21 ± 3.41	11.21 ± 3.87	−1.35	0.18
O	171.90 ± 38.86	176.79 ± 36.18	−0.67	0.51

Alpha level was set at *p* < 0.05

*SC* Self Care, *V* Vision, *L* Language, *M* Mobility, *W* Work, *UE* Upper Extremity, *T* Thinking, *P* Personality, *MD* Mood, *FR* Family Role, *SR* Social Role, *E* Energy, *O* Overall

**Table 6:** One-way ANOVA comparison of the Yoruba version of the Stroke-Specific Quality of Life 2.0 domains by age group

	Age groups					
SS-QoL Domains	<50 x̄ ±SD (*n* = 23)	50–59 x̄ ±SD (*n* = 40)	60–69 x̄ ±SD (*n* = 26)	≥ 70 x̄ ±SD (*n* = 11)	F-ratio	*p*-value
SC	16.91 ± 5.05	17.30 ± 5.17	18.04 ± 4.83	17.55 ± 5.24	0.216	0.881
V	13.61 ± 1.90	13.15 ± 2.53	14.12 ± 2.05	14.15 ± 12.05	1.281	0.285
L	20.48 ± 6.06	20.90 ± 4.70	22.62 ± 3.48	23.18 ± 3.22	1.572	0.201
M	21.43 ± 4.91	21.18 ± 6.45	21.46 ± 5.35	20.70 ± 8.23	0.047	0.986
W	7.74 ± 3.49	9.10 ± 3.66	8.38 ± 3.62	8.40 ± 4.88	0.667	0.575
UE	16.65 ± 4.51	16.30 ± 6.24	17.50 ± 5.47	15.60 ± 6.10	0.360	0.782
T	11.96 ± 3.28	9.97 ± 4.41	11.27 ± 3.30	11.18 ± 3.74	1.445	0.235
P	10.91 ± 3.92	9.68 ± 3.54	10.46 ± 3.24	11.40 ± 3.60	0.968	0.411
MD	19.39 ± 4.36	18.55 ± 5.62	20.35 ± 4.81	20.60 ± 4.22	0.882	0.453
FR	9.35 ± 3.90	10.15 ± 4.44	11.15 ± 3.68	10.27 ± 5.16	0.756	0.521
SR	10.91 ± 5.33	13.10 ± 6.41	14.46 ± 7.02	11.73 ± 6.80	1.387	0.251
E	10.26 ± 3.49	10.60 ± 3.61	10.92 ± 3.43	10.55 ± 4.63	0.134	0.940
O	169.61 ± 28.40	169.98 ± 38.41	180.73 ± 35.07	172.45 ± 45.47	0.552	0.648

Alpha level was set at *p* < 0.05

*SC* Self Care, *V* Vision, *L* Language, *M* Mobility, *W* Work, *UE* Upper Extremity, *T* Thinking, *P* Personality, *MD* Mood, *FR* Family Role, *SR* Social Role, *E* Energy, *O* Overall

The details for item-domain correlations (discriminant validity) for SS-QoL (Y) are presented in Table 7. The result showed that item-domain correlations were comparable within each domain of SS-QoL (Y). Items in ten out of the twelve domains had correlation scores >0.7 (*r* ranged from 0.711–0.920) with their hypothesized domains. The remaining two domains (Self-care and Mood) had item-domain correlation of <0.7 for a few of their items (*r* ranged from 0.594 to 0.630). Most items had correlation scores greater than 0.20 with other than their hypothesized domains except a few items (Table 7).

**Table 7:** Item-domain correlations (discriminant validity) of the Yoruba version of the Stroke Specific Quality of Life 2.0 (*n* = 100)

SS-QoL Domain	SC	V	L	M	W	UE	T	P	MD	FR	SR	E
SC1	0.703	0.044	0.108	0.344	0.704	0.598	0.017	0.216	0.057	0.167	0.412	0.297
SC2	0.594	0.341	0.328	0.365	0.137	0.293	0.165	0.245	0.400	0.257	0.156	0.328
SC4	0.782	0.243	0.171	0.544	0.482	0.540	0.011	0.240	0.241	0.288	0.315	0.250
SC5	0.829	0.390	0.312	0.529	0.580	0.437	−0.030	0.166	0.180	0.226	0.286	0.151
SC8	0.763	0.388	0.355	0.575	0.382	0.466	0.115	0.157	0.444	0.434	0.341	0.404
V1	0.306	0.764	0.347	0.353	0.043	0.101	0.183	0.169	0.279	0.188	0.095	0.063
V2	0.239	0.720	0.409	0.267	0.017	0.080	0.149	0.156	0.223	0.186	0.014	0.032
V3	0.269	0.799	0.472	0.345	0.040	0.136	0.175	0.313	0.219	0.150	0.000	0.083
L2	0.297	0.454	0.920	0.333	0.246	0.074	0.286	0.335	0.381	0.252	0.190	0.027
L3	0.312	0.521	0.885	0.436	0.147	0.097	0.369	0.378	0.542	0.358	0.114	0.145
L5	0.240	0.437	0.880	0.357	0.084	0.030	0.446	0.400	0.477	0.402	0.171	0.090
L6	0.316	0.471	0.908	0.426	0.198	0.123	0.432	0.497	0.492	0.371	0.217	0.158
L7	0.218	0.386	0.892	0.357	0.124	0.052	0.388	0.370	0.508	0.391	0.177	0.125
M1	0.554	0.259	0.262	0.776	0.525	0.425	0.162	0.206	0.301	0.383	0.409	0.381
M4	0.595	0.295	0.328	0.881	0.466	0.339	0.239	0.323	0.369	0.473	0.410	0.374
M6	0.624	0.282	0.336	0.803	0.608	0.486	0.224	0.347	0.338	0.312	0.372	0.397
M7	0.353	0.328	0.323	0.760	0.314	0.357	0.431	0.236	0.393	0.422	0.292	0.496
M8	0.573	0.434	0.430	0.788	0.355	0.316	0.246	0.299	0.386	0.412	0.254	0.325
M9	0.426	0.390	0.449	0.799	0.364	0.365	0.188	0.296	0.372	0.351	0.306	0.383
W1	0.571	0.064	0.148	0.447	0.914	0.595	0.112	0.262	0.006	0.199	0.444	0.295
W2	0.538	0.030	0.138	0.380	0.889	0.545	0.080	0.201	0.066	0.181	0.412	0.273
W3	0.554	0.056	0.179	0.461	0.875	0.551	0.113	0.266	0.244	0.226	0.551	0.365
UE1	0.433	0.138	0.256	0.276	0.616	0.703	0.187	0.285	0.197	0.189	0.395	0.308
UE2	0.502	0.086	0.044	0.485	0.556	0.715	0.013	0.289	0.238	0.249	0.399	0.440
UE3	0.560	0.093	0.074	0.400	0.465	0.770	0.171	0.240	0.292	0.195	0.347	0.389
UE4	0.357	0.196	−0.015	0.275	0.359	0.730	0.098	0.057	0.008	0.041	0.095	0.210
UE5	0.479	0.182	0.143	0.386	0.343	0.785	0.167	0.265	0.227	0.147	0.206	0.271
T2	0.047	0.259	0.348	0.245	0.125	0.132	0.884	0.441	0.459	0.407	0.256	0.300
T3	0.026	0.179	0.347	0.221	0.095	0.114	0.918	0.508	0.517	0.450	0.387	0.249
T4	0.023	0.180	0.502	0.283	0.077	0.081	0.787	0.522	0.564	0.365	0.228	0.220
P1	0.236	0.233	0.480	0.334	0.243	0.178	0.513	0.828	0.568	0.425	0.336	0.244
P2	0.195	0.197	0.392	0.222	0.103	0.022	0.397	0.733	0.444	0.378	0.253	0.134
P3	0.127	0.080	0.210	0.141	0.290	0.308	0.478	0.800	0.295	0.251	0.460	0.275
MD2	0.263	0.250	0.376	0.256	0.108	0.182	0.365	0.387	0.729	0.553	0.370	0.319
MD3	0.067	0.327	0.468	0.284	−0.181	0.026	0.449	0.335	0.630	0.469	0.099	0.233
MD6	0.274	0.140	0.380	0.321	0.171	0.269	0.448	0.518	0.721	0.579	0.594	0.398
MD7	0.145	0.188	0.381	0.289	0.089	0.100	0.425	0.406	0.772	0.606	0.399	0.478
MD8	0.371	0.348	0.394	0.403	0.119	0.117	0.343	0.295	0.608	0.390	0.231	0.291
FR5	0.280	0.322	0.515	0.387	0.022	0.040	0.488	0.412	0.673	0.731	0.392	0.228
FR7	0.374	0.265	0.341	0.480	0.181	0.162	0.344	0.389	0.697	0.838	0.524	0.407
FR8	0.307	0.096	0.280	0.287	0.368	0.234	0.353	0.318	0.409	0.826	0.783	0.257
SR1	0.225	0.069	0.136	0.140	0.593	0.348	0.274	0.420	0.302	0.494	0.801	0.325
SR4	0.262	0.069	0.230	0.288	0.423	0.284	0.156	0.303	0.364	0.593	0.816	0.272
SR5	0.403	0.031	0.142	0.156	0.295	0.315	0.435	0.407	0.463	0.609	0.800	0.352
SR6	0.302	0.023	0.211	0.271	0.424	0.311	0.116	0.388	0.382	0.512	0.778	0.329
SR7	0.376	0.025	0.201	0.304	0.452	0.338	0.327	0.349	0.396	0.621	0.859	0.313
E1	0.252	0.083	0.156	0.352	0.247	0.190	0.392	0.385	0.563	0.401	0.438	0.716
E2	0.312	0.015	0.123	0.497	0.411	0.489	0.374	0.378	0.408	0.438	0.510	0.711
E3	0.293	0.206	0.263	0.470	0.335	0.282	0.181	0.269	0.351	0.281	0.306	0.780

*SC* Self Care, *V* Vision, *L* Language, *M* Mobility, *W* Work, *UE* Upper Extremity, *T* Thinking, *P* Personality, *MD* Mood, *FR* Family Role, *SR* Social Role, *E* Energy

Intraclass correlation coefficient between participants’ score on SS-QoL (Y) scale on two occasions was significant for the overall score (0.74) and for the domain scores (0.47 to 0.81). The ICC was lowest (0.47) for family role domain and showed highest values (0.81) on two domains (language and upper extremity). ICC was above 0.7 in six domains, above 0.6 in three domains and between 0.47 and 0.55 in three domains (Table 8). Cronbach’s alpha values for domains of SS-QoL (Y) ranged from 0.61 to 0.82 (Table 9).

**Table 8:** Intra-class Correlation of scores on Yoruba version of SS-QoL 2.0 Scale on two occasions

		95% CI		
Domains	ICC	Lower bound	Upper bound	*p*-value
Self Care	0.78	0.66	0.86	0.001*
Vision	0.68	0.32	0.67	0.001*
Language	0.81	0.70	0.88	0.01*
Mobility	0.78	0.66	0.86	0.001*
Work	0.76	0.63	0.84	0.001*
Upper Extremity	0.81	0.71	0.88	0.001*
Thinking	0.74	0.61	0.83	0.001*
Personality	0.69	0.54	0.80	0.001*
Mood	0.60	0.42	0.73	0.001*
Family Role	0.47	0.26	0.64	0.001*
Social Role	0.55	0.35	0.69	0.001*
Energy	0.52	0.33	0.68	0.001*
Overall	0.74	0.61	0.83	0.001*

*ICC* Intra-class Correlation, *CI* Confidence Interval

*indicates significant correlation (*p* < 0.05)

**Table 9:** Cronbach’s alpha value for domains of Yoruba version of SS-QoL 2.0

Domains	Number of items	Mean ± SD	Cronbach’s alpha
Self Care	5	17.43 ± 5.28	0.77
Vision	3	13.62 ± 2.20	0.61
Language	5	21.48 ± 4.78	0.75
Mobility	6	21.05 ± 5.50	0.77
Work	3	8.44 ± 3.85	0.78
Upper Extremity	5	16.49 ± 5.69	0.82
Thinking	3	10.89 ± 3.76	0.62
Personality	3	10.21 ± 3.66	0.71
Mood	5	19.26 ± 4.77	0.72
Family Role	3	10.21 ± 3.86	0.70
Social Role	5	11.26 ± 5.21	0.70
Energy	3	10.58 ± 3.64	0.62

## Discussion

This study assessed the convergent, divergent, discriminant and known group validity, test-retest reliability and internal consistency of the SS-QoL (Y). The mean age of stroke survivors who participated in this study was 55.27 ± 12.34 years with median time since stroke onset of 11 months. These findings support the earlier reports that stroke occurs more frequently in middle age [15, 22, 29, 33–37]. Majority (68%) of the stroke survivors in this study were in the age group of 50 years and above. From the aforementioned reports, it can be stated that stroke majorly affect people in their middle age. Although stroke risk increases with age, strokes can occur and do occur at any age [38] while time from stroke varies considerably among the survivors. This may be attributed to factors such as presence of co-morbidities, quality of care and access to care and rehabilitation, racial and ethnic disparities, perceived social support and psychological state among other factors [1]. Sex distribution of participants in this study also indicated a male preponderance and this is in accordance with findings from previous studies [15, 22, 29, 34–36, 39]. Majority of the stroke survivors in this study had ischaemic stroke while more than half had right hemiplegia. This is similar to report of Andersen et al. [40] that ischaemic stroke is more frequent than haemorrhagic stroke. Similarly, left hemispheric ischaemic stroke presenting with right hemiplegia has been reported to be more frequent than right hemispheric cases [41] as reported by higher proportion of right hemiplegia/hemiparesis reported in this study.

The Yoruba version of WHOQoL-BREF was used to test the convergent validity of the SS-QoL (Y). The overall scores of SS-QoL (Y) showed fair to moderate significant correlation with all the four domains of WHOQoL-BREF. The significant correlations between similar domains of SS-QoL (Y) and WHOQoL-BREF may imply that the SS-QoL (Y) scale fairly captured relevant QoL domains which are also assessed by the WHOQoL-BREF.

Similar to the findings of this study, Boosman et al. [34] found significant correlation between the cognitive Failures Questionnaire (CFQ), Life Satisfaction-9 (LiSat-9), Hospital Anxiety Depression Scale (HADS) and corresponding SS-QoL 2.0 domain scores. The SS-QoL 2.0 scale has been compared with various other measures with varying findings. Muus et al. [13] reported moderate to excellent correlation (r ranging from 0.37 to 0.88) between the domains of Danish version of SS-QoL 2.0 and the Barthel Index and National Institute of Health Stroke Scale. Similar to the findings of the present study, Boosman et al. [34] reported that SS-QoL 2.0 scores showed weak to moderate correlations (0.24–0.32) with the Glasgow Outcome Scale and moderate to strong correlations (0.35–0.72) between SS-QoL 2.0 scores and CFQ, LiSat-9 and HADS. Lin et al. [35] also reported weak to moderate correlations (r ranging from −0.04 to 0.52) between the domains of the SS-QoL 2.0 and Fugl-Meyer Assessment, Functional Independent Measure, and Frenchay Activities Index with excellent correlation (*r* = 0.65) only between the SS-QoL 2.0 Self-Care domain and Functional Independent Measure. Hakverdioglu and Khorshid [17] reported moderate correlation between the mean overall score of the Turkish version of SS-QoL and the mean domain scores as well as between the SS-QoL domains scores and the mean scores of the sub- dimensions of SF-36. Moderate correlation was also found between the total mean score of the Turkish version SS-QoL and the mean score of Katz Index of ADL. The findings in the present study are consistent with findings in the reported studies. Although the correlation between the four domains of WHOQoL-BREF and similar SS-QoL domains is significant, the correlation coefficients are low (*r* < 0.4). Certain factors may be responsible for this. Previous studies that compared the SS-QoL with generic scales used more than one scale for comparison such that most related domains on the SS-QoL and the other scales were compared [12, 13, 17, 42, 43]. In the current study however, the domains of WHOQoL-BREF only were used to compare the domains of SS-QoL 2.0 and this may not allow for comparison with most related domains as obtained in previous studies. Although some of these studies also reported weak correlation with some of the SS-QoL 2.0 domains [13, 42, 43], this was explained by the elapsed time between the stroke event and the administration of the questionnaire [42]. This may also be applicable to our study in which time since stroke onset was more than 1 month prior to the administration of the questionnaires. The process of social adaptation has been reported to be a possible factor that can cancel out supposed differences in QoL. This fact can also explain in like manner the apparent weak but significant correlation between the generic measure (WHOQoL-BREF) and a specific measure (SS-QoL 2.0) [13, 42]. The items in each domain of SS-QoL 2.0 were structured in a specific and simplified manner that addresses stroke-related problems, but this is not so for the generic WHOQoL-BREF. For example, two items on physical health domain of WHOQoL-BREF “How well are you able to get around?” and “How satisfied are you with your transport?” are related to mobility domain of SS-QoL2.0 which comprised six different items. Social relationship domain of WHOQoL-BREF has three items which is like a summary of a total of eight items on the family role and social role domains of SS-QoL2.0. Language and vision domains of SS-QoL 2.0 comprise five and three items respectively, but no items on the WHOQOL-BREF assess any of these domains. The differences in the structure of the items on the two scales as exemplified above may also explain the weak correlations between the compared domains.

Dissimilar domains on Yoruba versions of SS-QoL and WHOQoL-BREF were also compared to assess divergent validity of the SS-QoL (Y). Poor to fair correlation (*r* ranged from 0.035 to 0.366) were demonstrated between dissimilar domains of the two scales. A number of these domains however demonstrated significant correlation. Studies have shown that some types of adaptation and coping strategies are adopted by stroke survivors in order to resume pre-stroke life [33, 44, 45], some of which may be more effective in coping with the consequences of the stroke than others [33, 44]. Significant correlation was found between the self-care domain of SS-QoL (Y) and all the domains of Yoruba version of WHOQoL-BREF except environment domain. This may be explained in terms of the limitation in physical functioning, a common consequence of hemiplegia or hemiparesis which might have been adapted to by the stroke survivors in this study. It might also be that they have devised alternative means of self-care and coping strategy such that their physical health, psychological health and social relationship are no longer affected negatively by the condition. However, the physical disability that is usually associated with stroke might not have been overcome by the architecture of their environment and as a result, still interfere negatively with their quality of life. Language domain of SS-QoL (Y) also correlated weakly but significantly with PH and PSH domains of Yoruba version of WHOQoL-BREF. The frustration that is usually associated with aphasia or speech disturbances in some stroke survivors might have resolved in the participants in this study, majority of whom have had stroke for 6 months and more. This may boost their psyche and thus explain the significant correlation between language and PSH domains of the SS-QoL and WHOQoL-BREF respectively. The vision, thinking and mood domains of SS-QoL (Y) correlated significantly with environment domain of WHOQoL-BREF. This may also be due to the use of coping strategies by the stroke survivors. As expected, the upper extremity and personality domains of SS-QoL (Y) did not correlate significantly with any of the domains of the Yoruba version WHOQoL-BREF. This may be because upper extremity and personality domains on SS-QoL are more specific to stroke survivors whereas the domains of WHOQoL-BREF are generic and did not capture these stroke specific domains enough. Family role and social role domains of the SS-QoL (Y) also correlated significantly with the PSH domain of WHOQoL-BREF. This may also be explained in terms of coping strategies that the stroke survivors might have learnt in their family and social functioning. Similarly, mobility, work, personality, mood and energy domains of SS-QoL (Y) demonstrated significant correlation with SoR domain of WHOQoL-BREF possibly as a consequence of adaptation and coping strategy engendered by the stroke to enable the survivors resume a normal life. The finding for the known-group validity of the SS-QoL (Y) indicated that there was no significant gender difference between the domains and overall scores. This is consistent with report of Xie et al. [1] and Zalihic et al. [46]. Male participants had higher mean score in work, upper extremity, thinking and family role domains while female participants had higher mean score in the remaining nine domains and the overall QoL score. Conversely, previous studies reported significantly lower post-stroke QoL in females and that females are more negatively affected in their QoL [47–49]. In these studies, QoL was assessed using different QoL scales, mode of administration and stroke patients in different settings from the current study, and these factors may be responsible for the observed differences in QoL and gender.

The finding of this study also showed that the SS-QoL domains were associated with age. The mean domain scores increased steadily across three out of the four age groups (<50 years, 50–59 years and 60–69 years) on self-care, language, family role, social role and energy domains and the overall score. A decline in mean scores was observed for the oldest age group (≥70 years) on self-care, mobility, upper extremity, family role and social role domains while steady increase in the mean overall score was observed across all age groups. The reported differences in the QoL scores among participants in the current study is not significant but has demonstrated that age of stroke survivors was an important factor that determines their HRQoL [50–53]. Psychological function appeared to be less affected by age as demonstrated by SS-QoL scores on personality, thinking and mood domains consistent with findings of Alonso-Moran et al. [52] and Jeste et al. [54].

The items on SS-QoL (Y) showed a high level of item-domain correlations (i.e., correlations of an item with its own domain). All items in the SS-QoL (Y) demonstrated strong correlations with its hypothesized domain than with domains measuring other concepts. For example, item L2 ‘Did you have trouble speaking? For example, get stuck, stutter, stammer or slur your words?’ had r value of 0.920 with its own domain, while the same item had r value of 0.297 with SC domain. The only exception was item SC1 (‘did you have trouble preparing food?’ Translated as ‘N jẹ́ o nìlò ìrànlọ́wọ́ láti wá oúnjẹ’) that correlated strongly with work domain. The item SC1 had correlation value of 0.703 with its own domain and 0.704 for work domain. The finding of this study shows that there was significant correlation between all the domains of the SS-QoL scale as well as between the overall score and each of the domains’ score. This finding of strong correlations of items with its own domain is consistent with reports of Muus et al. [13] and Cruz-Cruz et al. [42] and describes the relevance of the items in their respective domains and the discriminant validity of the SS-QoL (Y).

The ICCs between participants’ scores on two occasions for the overall and domains of the SS-QoL (Y) indicated moderate to high test-retest reliability. Furthermore, the moderate to high Cronbach’s alpha values imply internal consistency or homogeneity of the domains of the scale. This is comparable with internal consistency or interrelatedness of the domains of SS-QoL 2.0 scale reported by Hsueh et al. [9]. The findings are comparable with the reports from previous studies on different versions of SS-QoL 2.0 scale [4, 11, 13, 15, 17, 34, 42, 55, 56]. For example, Williams et al. [4] and Boosman et al. [34] reported excellent internal consistency for ischaemic stroke survivors and patients with aneurysmal subarachnoid haemorrhage respectively. Similarly, Kerber et al. [55] reported excellent test-retest reliability and internal consistency for SS-QoL 2.0 scale in Mexican American English speaking population. Danish version was reported to have moderate to excellent test retest reliability and excellent internal consistency by Muus et al. [13]. The Brazilian version of SS-QoL 2.0 was reported by Lima et al. [15] to have excellent test-retest reliability while the Spanish version of SS-QoL 2.0 good to excellent test retest reliability and internal consistency [11, 42]. Harkverdioglu and Khorshid [17] reported excellent test-retest reliability and excellent internal consistency for Turkish version of SS-QoL 2.0, while the Chinese version of SS-QoL 2.0 demonstrated good to excellent internal consistency according to Wong et al. [56].

This study has some limitations that may need to be considered in interpreting and generalizing its findings. First, the generalizability of this study may be limited to stroke survivors in urban and semi urban communities. This is because stroke survivors from only departments of physiotherapy in health facilities within each of the capital city and other major cities of the south-western states of Nigeria were included. Second, there are possible differences in psychometric properties in patient-reported QOL outcomes due to the modes of administration [57] thus further research may be needed to study psychometric properties of the SS-QoL (Y) using different modes of administration such as paper-and-pencil administration at home, via the mail and telephone interview. Participants in this study received different rehabilitation programs throughout the duration of the study; further research is needed to assess the psychometric properties of the SS-QoL (Y) for specific treatment programs on larger samples to provide further insights into the psychometric properties of the SS-QoL (Y) in particular situations. Lastly, the SS-QoL (Y) was validated using the Yoruba version of WHOQoL-BREF in this study, other Yoruba outcome measures with the advantage of assessing domains relevant to stroke, should be used to compare the domains of SS-QoL (Y) in order to further ascertain its validity with other established measures.

## Conclusion

The test-retest reliability, internal consistency, discriminant and known-group validity of the SS-QoL(Y) are adequate. The scale fairly and significantly captured all related domains on the Yoruba version of WHOQoL-BREF which indicates acceptable convergent validity. The SS-QoL(Y) is therefore a valid and reliable measure recommended for assessing health-related quality of life among Yoruba stroke survivors.

## Additional files

Additional file 1: Yoruba Version Of SS-QoL 2.0. (DOCX 31 kb)

Additional file 2: Yorùbá Version Of Whoqol-Bref. (DOCX 19 kb)

## Abbreviations

ADL: Activity of daily living
AEV: Adapted english version
CFQ: Cognitive failure questionnaire
CI: Confidence interval
E: Energy
Env: Environment
FR: Family role
GOS: Glasgow outcome scale
HADS: Hospital anxiety and depression scale
HRQoL: Health related quality of life
ICC-Intra: Class correlation coefficient
L: Language
LiSAT: Life satisfaction questionnaire
M: Mobility
MD: Mood
O: Overall
P: Personality
PH: Physical health
PSH: Psychological health
QoL: Quality of life
SC: Self care
SoR: Social relationship
SPSS: Statistical package for social sciences
SR: Social role
SS-QoL 2.0 (Y): Yoruba version of stroke-specific quality of life scale version 2.0
SS-QoL 2.0: Stroke-specific quality of life scale version 2.0
T: Thinking
UE: Upper extremity
V: Vision
W: Work
WHOQoL-BREF: World health organisation quality of life short form
YTV: Yoruba translated version

## References

[1] Xie, J; Wu, EQ; Zheng, Z; Croft, JB; Greenlund, KJ; Mensah, GA; Labarthe, DR. Impact of Stroke on Health-Related Quality of Life in the Non-institutionalized Population in the United States. Stroke. 2006; doi: 10.1161/01.STR.0000240506.34616.10. 10.1161/01.STR.0000240506.34616.10 16946158

[2] Owolabi MO. Determinants of health-related quality of life in Nigerian stroke survivors. Trans R Soc Trop Med Hyg. 2008;102(12):1219–25. doi: 10.1016/j.trstmh.2008.05.003. Epub 2008 Jun 16 10.1016/j.trstmh.2008.05.003 18556034

[3] Salter KL, Moses MB, Foley NC, Teasell RW (2008). Health-related quality of life after stroke: what are we measuring?. Int J Rehabil Res.

[4] Williams LS, Weinberger M, Harris LE, Clark DO, Biller J (1999). Development of a stroke- specific quality of life scale. Stroke.

[5] Muus I, Ringsberg KC (2005). Stroke -specific quality of life scale: Danish adaptation and a pilot study for testing psychometric properties. Scand J Caring Sci.

[6] Verbunt JA, Seelen HAM, Ramos FP, Michielsen BHM, Wetzelaer WL, Moennekens M (2008). Mental practice-based rehabilitation training to improve arm function and daily activity performance in stroke patients: a randomized clinical trial. BMC Neurol.

[7] Chou PC, Chu HY, Lin JG (2009). Effects of electroacupuncture treatment on impaired cognition and quality of life in Taiwanese stroke patients. J Altern Complement Med.

[8] Carod-Artal FJ, Egido JA (2009). Quality of life after stroke: the importance of a good recovery. Cerebrovasc Dis Disease.

[9] Hsueh I, Jeng J, Lee Y, Sheu C, Hsieh C (2011). Construct validity of quality of life stroke questionnaire on Ischaemic stroke patients. Arch Phys Med Rehabil.

[10] Saladin LK (2000). Measuring quality of life post stroke. Copyright neurology report htm. Provided by pro quest information and learning company.

[11] Fernández-Concepción O, Verdecia-Fraga R, Alvarez-González MA, Román, Pastoriza Y, Ramírez-Pérez E *.* Stroke-specific quality of life scale (ECVI-38): an evaluation of its acceptance, reliability and validity]. Rev Neurol 2005;41(7):391-8. 16193444

[12] Ewert, Stucki (2007). Validity of the SS-QOL in Germany and in survivors of hemorrhagic or ischemic stroke. Neurorehabil Neural Repair.

[13] Muus I, Williams LS, Ringsberg KC (2007). Validation of the stroke-specific quality of life scale: test of reliability and validity SS-QOL-DK. Clin Rehabil.

[14] Kartsona A, Hilari K (2007). Quality of life in aphasia: Greek adaptation of the stroke and aphasia quality of life scale-39 item (SAQoL-39). Eura Medicophys.

[15] Lima RCM, Teixeira-Salmela LF, Magalhaes LC, Gomes-Neto M (2008). Psychometric properties of the Brazilian version of the stroke specific quality of life scale: application of the Rasch model. Rev Bras Fisioter.

[16] Wang Y, Ma J, Li J et al. The study on reliability, validity and responsiveness of the Chinese version of Stroke-specific Quality of Life. http://en.cnki.com.cn/Journal_en/E-EO6-LNXG-2003-06htm. Accessed on 2 March, 2016.

[17] Hakverdioğlu Yönt G, Khorshid L (2012). Turkish version of the stroke-specific quality of life scale. Int Nurs Rev.

[18] Kiran S, Krishnan G. Stroke and aphasia quality of life scale in Kannada-evaluation of reliability, validity and internal consistency. Available from: http://www.annalsofian.org/text.asp?2013/16/3/361/116932. Accessed on 23 Jan. 2015. 10.4103/0972-2327.116932 PMC3788281 24101817

[19] Mahmoodi M, Safari A, Vossoughi M, Golbon-Haghighi F, Kamali-Sarvestani M, Ghaem H, Borhani-Haghighi A (2015). Stroke-specific quality of life questionnaire: test of reliability and validity of the Persian version. Iran J Neurol.

[20] Raju R, Krishnan G (2015). Adaptation and validation of stroke-aphasia quality of life (SAQoL-39) scale into Malayam. Ann Indian Acad Neurol.

[21] Mitra I, Krishnan G (2015). Adaptation and validation of stroke-aphasia quality of life scale (SAQoL-39) to Hindi. Ann Indian Acad Neurol.

[22] Akinpelu A (2012). O; Odetunde M O; Odole C a. Cross-cultural adaptation and initial validation of stroke-specific quality of life scale into Yoruba language. Int J Rehabil Res.

[23] de Morton NA, Davidson M, Keating KL (2010). Validity, responsiveness and the minimal clinically important difference for the de Morton mobility index (DEMMI) in an older acute medical population. BMC Geriatr.

[24] Laake P, Olsen BR, Benestad HB (2007). Research methodology in the medical and biological sciences.

[25] Gjersing L**,** Caplehorn JRM, Clausen, T. Cross-cultural adaptation of research instruments: language, setting, time and statistical considerations. BMC Med Res Methodol 2010. doi:10.1186/1471-2288-10-13**.** 10.1186/1471-2288-10-13 PMC2831007 20144247

[26] Beaton DE, Claire MD, Guillemin FMD (2000). Guidelines for the process of cross-cultural adaptation of self-report measures. Spine.

[27] Schmidt B (2003). Current issues in cross-cultural quality of life instrument development. Arch Phys Med Rehabil.

[28] Mamora O. Yoruba at home and in Diaspora: partnering for development. Nigeria world 2006. http://nigeriaworld.com/cgibin/search/search.pl?Realm=Nigeriaworldiiki&match=1&Terms=Olorunnimbe+Mamora&Rank=1. Accessed 29 April 2012.

[29] Lin K; Fu T; Wu C and Hsieh C. Assessing the Stroke-Specific Quality of Life for Outcome Measurement in Stroke Rehabilitation: Minimal Detectable Change and Clinically Important Difference. Health Qual Life Outcomes 2011. doi:10.1186/1477-7525-9-5. 10.1186/1477-7525-9-5 PMC3034658 21247433

[30] WHOQoL Group (1998). Development of the WHOQoL-BREF quality of life assessment. Psychol Med.

[31] Akinpelu AO, Maruf FA, BOA A (2006). Validation a Yoruba translation of the World Health Organisation’s quality of life scale-short form among stroke survivors in south western Nigeria. Afr J Med Med Sci.

[32] Bowling A: Mode of questionnaire administration can have serious effects on data quality. J Public Health 2005; 27(3):281-292. DOI:https://doi/10.1093/pubmed 10.1093/pubmed/fdi031 15870099

[33] Lynch EB, Butt Z, Heinemann A, Victorson D, Nowinski CJ, Perez L, Cella D (2008). A qualitative study of quality of life after stroke: the importance of social relationships. J Rehabil Med.

[34] Boosman H, Passier PECA, Visser-Meily JMA, Rinkel GJE, Post MWM (2010). Validation of the stroke-specific quality of life scale (SS-QOL 2.0) in patients with aneurismal subarachnoid haemorrhage. J Neurol Neurosurg Psychiatry.

[35] Lin KC, Fu T, Wu CY, Hsieh YW, Chen CL, Lee PC (2010). Psychometric comparisons of the stroke impact scale 3.0 and stroke-specific quality of life scale. Qual Life Res.

[36] Chen H, Wu C, Lin K, Li M, Yu H (2012). Validity, reliability and responsiveness of a short version of the stroke-specific quality of life scale in patients receiving rehabilitation. J Rehabil Med.

[37] Hamzat TK, Olaleye OA, Akinwumi OB (2014). Functional ability, community reintegration and participation restriction among community dwelling female stroke survivors in Ibadan. Ethiop J Health Sci.

[38] Smajlović D. Strokes in young adults: epidemiology and prevention. Vasc Health Risk Manag. 2015;11:157–64 doi:10.2147/VHRM.S53203. 10.2147/VHRM.S53203 PMC4348138 25750539

[39] Falcone G; and Chong JY. Geriatr Aging 2007:10(08):497–500.

[40] Andersen KK; Olsen TS; Dehlendorff C; Kammersgaard LP. Hemorrhagic and ischemic strokes compared: stroke severity, mortality, and risk factors. Stroke 2009. doi:10.1161/STROKEAHA.108.540112. 10.1161/STROKEAHA.108.540112 19359645

[41] Hedna VS; Bodhit AN; Ansari S; Falchook AD; Stead L; Heilman KM, Waters FM Hemispheric differences in Ischaemic Stroke: Is left hemisphere stroke more common. J Clin Neurol. 2013l doi: 10.3988/jcn.2013.9.2.2.97 10.3988/jcn.2013.9.2.97 PMC3633197 23626647

[42] Cruz-Cruz C, Martinez-Nuñez JM, Perez ME, Pharm D, Kravzov-Jinich J, Ríos-Castañeda C, Altagracia-Martinez M. Evaluation of the Stroke-Specific Quality-of-Life (SS-QoL) Scale in Mexico: A Preliminary Approach. Value Health Reg Issues 2013. doi: 10.1016/j.vhri.2013.04.002.%20Accessed%20on16/4/2015 10.1016/j.vhri.2013.04.002 29702776

[43] Li JT, Wang YL, Yu J H. The study on validity of the Chinese version of the Stroke specific Quality of Life in the south of Ji. Journal of Brain and Nervous Diseases 2007. Accessed on http://en.cnki.cn/Journal_en/E-E070-LYSJ-2007-03.htm. Accessed on 16/4/2015.

[44] Rochette A, Desrosiers J (2002). Coping with the consequences of a stroke. Int J Rehabil Res.

[45] Donnellan C, Havey D, Hickey A, O’Neill D (2006). Defining and quantifying coping strategies after stroke: a review. J Neurol Neurosurg Psychiatry.

[46] Zalihić A, Markotić V, Zalihić D, Mabić M (2010). Gender and quality of life after cerebral stroke. Bosn J Basic Med Sci.

[47] Gargano JW, Reeves MJ (2007). Sex differences in stroke recovery and stroke-specific quality of life. Result from a state-wide stroke registry inc. Stroke.

[48] Franzén-Dahlin Å, Laska AC. Gender differences in quality of life after stroke and TIA: a cross-sectional survey of out-patients. J Clin Nurs. 2012;21(15–16):2386-2391. 2012 DOI: 10.1111/j.1365-2702.2011. 04064.x. 10.1111/j.1365-2702.2011.04064.x 22788569

[49] Bushnell CD, Reeves MJ, Pan W, Prvu-Bettger J, Zimmer L, Olson D, Peterson E. Sex differences in quality of life after ischemic stroke. Neurology 2014; doi:10.1212/WNL.0000000000000208 PMCID: PMC4211921. 10.1212/WNL.0000000000000208 PMC4211921 24510493

[50] Nichols-Larsen DS, Clark PC, Zeringue A, Greenspan A, Blanton S. Factors Influencing Stroke Survivors’ Quality of Life During Subacute Recovery. http://stroke.ahajournals.org/content/strokeaha/36/7/1480.full.pdf. Accessed on 24 August 2017. 10.1161/01.STR.0000170706.13595.4f 15947263

[51] Badaru UM, Ogwumike OO, Adeniyi AF (2015). Quality of life of Nigerian stroke survivors and its determinants. Afr J Biomed Res.

[52] Alonso-Moran E, Nuno- Solinis R et al. Health-related quality of life and multimorbidity in community-dwelling telecare- assisted elders in the Basque Country. European Journal of Internal Medicine 2015; 26:169-175. 10.1016/j.ejim.2015.02.013 25704329

[53] Hong. Age Differences in health-related quality of life among South Korean elderly. Research & Reviews: J. Nurs. Health Sci. 2015. http//www.rroij.com/open-access/agedifferences-in-healthrelated-quality-of-life-among-southkorean-elderly.php?aid=64486. Accessed on 22 August 2017.

[54] Jeste DV, Savla GN (2013). Association between older age and more successful aging: critical role of resilience and depression. Am J Psychiatry.

[55] Kerber KA, Brown DL, Skolarus LE, Morgenstern LB, Smith MA, Garcia NM, Lisabeth LD.Validation of the 12-Item Stroke-Specific Quality of Life Scale in a Bi-ethnic Stroke Population. J Stroke Cerebrovasc Dis. 2012; doi:10.1016/j.jstrokecerebrovasdis.2012.08.011. 10.1016/j.jstrokecerebrovasdis.2012.08.011 PMC3552000 22995379

[56] Wong GK, Lam SW, Ngai K, Wong A, Poon WS, Mok V. Validation of the Stroke-specific Quality of Life for patients after aneurysmal subarachnoid haemorrhage and proposed summary sub-scores. J Neurol Sci. 2012. doi: 10.1016/j.jns.2012.06.025. 10.1016/j.jns.2012.06.025 22818113

[57] Gundy CM, Aaronson NK (2010). Effects of mode of administration (MOA) on the measurement properties of the EORTC QLQ-C30: a randomized study. Health Qual Life Outcomes.

