# Supplementary material for: Validity and reliability of a Nigerian-Yoruba version of the stroke-specific quality of life scale 2.0
Source: Health Qual Life Outcomes. 2017 Oct 19;15:205. doi: 10.1186/s12955-017-0775-9 (PMC5649048; doi:10.1186/s12955-017-0775-9)
Supplement: Supplementary file 1 — Yoruba Version Of SS-QoL 2.0. (DOCX 31 kb) [file 12955_2017_775_MOESM1_ESM.docx]

ADDITIONAL FILE 1: YORUBA VERSION OF SS-QoL 2.0

ÒDIWỌ̀N ÌGBÁYÉGBÁDÙN ÀWỌN ALÁÌSÀN RỌPÁ-RỌSẸ̀ (Akinpelu et al. 2012)

ÌTỌ́NISỌ́NÀ: A fẹ́ mọ bí àìsàn rọpá-rọsẹ̀ ṣe ṣe àkóbá fún àwọn akitiyan tàbí ìmọ́lára tí àìsàn náà máa n ṣe àkóbá fún ní ìgbà mìíràn.Ìbéèrè kọ̀ọ̀kan wá làti ṣe àyẹ̀wò ìrírí rẹ pẹ̀lú bí akitiyan tàbí ìmọ̀lára kọ̀ọ̀kan ṣe rí fún ọ láàrin ọ̀sẹ̀ kan sẹ́yin.Yí òdo ká nómbà tó bá se àpèjúwe ìdáhùn rẹ sí àwọn gbólóhùn kọọkaṇ. Fi máàkì sí gbolohuṇ kan àti ìdáhùn tí ó bá gbolohun yìí mu lórí ìlà kan náà.

LÁÀRIN Ọ̀SẸ̀ KAN SẸ́YÌN

|  | N kò le ṣe é rárá  1 | Pẹ̀lú ọ̀pọ̀lọpọ̀ ìṣòro  2 | Pẹ̀lú ìṣòro níwọ̀nba  3 | | Pẹ̀lú ìṣòro bíntín  4 | Láìsí ìṣòro rárá  5 |
| --- | --- | --- | --- | --- | --- | --- |
| SC1.N jẹ́ o nìlò ìrànlọ́wọ́ láti wá oúnjẹ | 1 | 2 | 3 | | 4 | 5 |
| SC2.N jẹ́ o nílò ìrànlọ́wọ́ lati jẹ oúnjẹ rẹ? Bí àpẹẹrẹ bí o bá fẹ́ bu oúnjẹ tàbí láti mi ounjẹ? | 1 | 2 | 3 | | 4 | 5 |
| SC3.N jẹ́ o nílò ìrànlọ́wọ́ láti wọ aṣọ sọ́rùn? Bí àpẹẹrẹ igba ti o ba fẹ wọ ibọsẹ tabi bata, nipa dide awọn bọtini asọ rẹ, lati ro iro, wọ buba, we gele tabi de fila? | 1 | 2 | 3 | | 4 | 5 |
| SC4.N jẹ́ o nílò ìrànlọ́wọ́ láti wẹ̀ tàbí ṣanra? | 1 | 2 | 3 | | 4 | 5 |
| SC5.Ṣé o nílò ìrànlọ́wọ́ nígbàtí o bá fẹ́ ṣe ìgbọ̀nsẹ̀ tàbí tọ̀? | 1 | 2 | 3 | | 4 | 5 |
| V1.N jẹ́ ó máa n ṣòro fún ọ láti wo ẹ̀rọ móhùnmáwòrán débi pé o ó fi gbádun nnkan tí ò n wò? | 1 | 2 | 3 | | 4 | 5 |
| V2.N jẹ́ o ní ìṣòro láti nawọ́ mú nnkan nítorí pé o kì í ríran dáadáa tó? | 1 | 2 | 3 | | 4 | 5 |
| V3.N jẹ́ o ní ìsòro láti rí nnkan láti apá kan sí èkejì? | 1 | 2 | 3 | | 4 | 5 |
| L1.N jẹ́ o ní ìṣòro nípa ọ̀rọ̀ sísọ? Bí àpẹẹrẹ n jẹ́ o máa n há tí o bá n sọ̀rọ̀? Ṣé o máa n ṣe àwítúnwí,ṣé o máa n kálòlò,n jẹ́ ọ̀rọ̀ rẹ máa n fà? | 1 | 2 | 3 | | 4 | 5 |
| L2.N jẹ́ o ní ìṣòro láti sọ̀rọ̀ gaara débi pé o ó le lo ẹ̀rọ tẹlifóònù? | 1 | 2 | 3 | | 4 | 5 |
| L3.N jẹ́ àwọn ènìyàn máa n ní ìṣòro láti gbọ́ ọ ní àgbọ́yé? | 1 | 2 | 3 | | 4 | 5 |
| L4.N jẹ́ o máa n ní ìṣòro láti ṣe àwárí àwọn ọ̀rọ̀ tóo ba fẹ́ẹ́ lò? | 1 | 2 | 3 | | 4 | 5 |
| L5.N jẹ́ o máa n ní láti ṣe àsọtúnsọ ọ̀rọ̀ kí àwọn ènìyàn tó ó gbọ́ ọ ní  àgbọ́yé? | 1 | 2 | 3 | | 4 | 5 |
|  | N kò le ṣe é rárá  1 | Pẹ̀lú ọ̀pọ̀lọpọ̀ ìṣòro  2 | Pẹ̀lú ìṣòro níwọ̀nba  3 | Pẹ̀lú ìṣòro bíntín  4 | | Láìsí ìṣòro rárá  5 |
| M1.N jẹ́ o ní ìṣòro láti rìn? | 1 | 2 | 3 | 4 | | 5 |
| M2.Ṣé o kìi fẹ́ẹ́ ṣubú nígbàtí o bá bẹ̀rẹ̀ tàbí bí o bá fẹ́ẹ́ mú nnkan? | 1 | 2 | 3 | 4 | | 5 |
| M3.Ṣé o máa n ní ìṣòro láti gun àtẹ̀gùn ilé? | 1 | 2 | 3 | 4 | | 5 |
| M4.Ṣé o ní láti máa dúró simi ju bí o ti fẹ́ lọ nígbàtí o bá n rìn tàbí bí o bá wà lóri i kẹ̀kẹ́ àfirìn fun aláìsàn? | 1 | 2 | 3 | 4 | | 5 |
| M5.N jẹ́ o ní ìṣòro láti dádúró? | 1 | 2 | 3 | 4 | | 5 |
| M6.N jẹ́ o máa n ní íṣòro nígbàtí o bá fẹ́ẹ́ dìde lórí àga? | 1 | 2 | 3 | 4 | | 5 |
| W1.N jẹ́ o ní ìṣòro láti ṣe iṣẹ́ inú ilé àti àyíká lé? | 1 | 2 | 3 | 4 | | 5 |
| W2.N jẹ́ o ní ìṣòro láti parí àwọn iṣẹ́ tí o bẹ̀rẹ̀? | 1 | 2 | 3 | 4 | | 5 |
| W3.N jẹ́ o ní ìṣòro láti ṣe àwọn iṣẹ́ tí o ti n ṣe rí? | 1 | 2 | 3 | 4 | | 5 |
| UE1.N jẹ́ ó ni ọ́ lára láti kọ tàbí tẹ ìwé? | 1 | 2 | 3 | 4 | | 5 |
| UE2.N jẹ́ ó nira fún ọ láti wọ ìbọ̀sẹ̀, pátá tàbí ṣòkòtò? | 1 | 2 | 3 | 4 | | 5 |
| UE3.N jẹ́ ó nira fún ọ láti de bọ́tìnì aṣọ rẹ? | 1 | 2 | 3 | 4 | | 5 |
| UE4.N jẹ́ ó nira fún ọ láti de síìpù aṣọ rẹ? | 1 | 2 | 3 | 4 | | 5 |
| UE5.N jẹ́ ó ṣòro fún ọ láti ṣí ìdérí ìgò tàbí kẹ́ẹ̀gì? | 1 | 2 | 3 | 4 | | 5 |

Àwọn ìbéèrè ìsàlẹ̀ yìí wà láti mọ̀ bí o ṣe fara mọ́ tàbí lòdì sí àwọn gbólóhùn wọ̀nyìí tí ó dá lórí àìsàn rọpá-rọsẹ̀ tó. Yí òdo ká nómbà tó bá se àpèjúwe ìdáhùn rẹ sí àwọn gbólóhùn kọọkaṇ. Fi máàkì sí gbólóhùṇ kan àti ìdáhùn tí ó bá gbólóhùn yìí mu lórí ìlà kan náà.

LAARIN ỌSẸ KAN SẸYIN

|  | Mo fara mọ́ ọ gidigidi  1 | Mo fara mọ́ ọ níwọ̀nba  2 | N kò fara mọ́ ọ bẹ́ẹ̀ni n kò lòdì sí i  3 | Mo lòdì síi níwọ̀nba  4 | Mo lòdì sii pátá pátá  5 |
| --- | --- | --- | --- | --- | --- |
| T1.Ó ṣòro fún mi láti f’ọkàn sí nnkan pẹ́ títí | 1 | 2 | 3 | 4 | 5 |
| T2.Mo ní ìṣòro láti rántí nnkan | 1 | 2 | 3 | 4 | 5 |
| T3.Mo ní láti máa kọ nnkan sílẹ̀ kí n tóó le rántí wọn | 1 | 2 | 3 | 4 | 5 |
| P1.Mo máa n kanra | 1 | 2 | 3 | 4 | 5 |
| P2.N kò ní sùúrù pẹ̀lú àwọn ẹlòmííràn | 1 | 2 | 3 | 4 | 5 |
|  | | | | | |
|  | Mo fara mọ́ ọ gidigidi  1 | Mo fara mọ́ ọ níwọ̀nba  2 | N kò fara mọ́ ọ bẹ́ẹ̀ni n kò lòdì sí i  3 | Mo lòdì síi níwọ̀nba  4 | Mo lòdì sii pátá pátá  5 |
| P3.Ìrínisí mi ti yí padà | 1 | 2 | 3 | 4 | 5 |
| MD1.Mo ní ìrẹ̀wẹ̀sì ọkàn nípa ọjọ́ ọ̀la mi | 1 | 2 | 3 | 4 | 5 |
| MD2.N kò nífẹ̀ẹ́ láti mọ̀ nípa àwọn ẹ̀lómííràn tàbí ohunkóhun | 1 | 2 | 3 | 4 | 5 |
| FR1.N kò dara pọ̀ pẹ̀lú àwọn ẹbí mi láti ṣeré | 1 | 2 | 3 | 4 | 5 |
| FR2.Mo rí ara mi gẹ́gẹ́ bí ìnira fún àwọn ẹbí mi | 1 | 2 | 3 | 4 | 5 |
| FR3.Àìlera mi ṣe ìdíwọ́ fún ìgbé ayé mi | 1 | 2 | 3 | 4 | 5 |
| SR1.N kìí lọ sóde tó bí m bá ṣe fẹ́ | 1 | 2 | 3 | 4 | 5 |
| SR2.Àkókò tí mò n lò fún ìdárayá àti ìnàjú kéré ju bí m bá ṣe fẹ́ lọ | 1 | 2 | 3 | 4 | 5 |
| SR3.N kìí lè rí púpọ̀ nínú àwọn ọ̀rẹ́ mi tó bí m bá ṣe fẹ́ | 1 | 2 | 3 | 4 | 5 |
| SR4.Mo ní ìbálòpọ̀ kéré ju bí m bá ṣe fẹ́ | 1 | 2 | 3 | 4 | 5 |
| SR5.Àìlera mi n ṣe ìdíwọ́ fún ìgbé ayé tí m bá gbé l’áwùjọ | 1 | 2 | 3 | 4 | 5 |
| MD3.Mo máa n fà sẹ́yìn láti bá àwọn ẹlòmííràn ṣe | 1 | 2 | 3 | 4 | 5 |
| MD4.Ìfọkàntán tí mo ní nínú araà mi kò tó nnkan | 1 | 2 | 3 | 4 | 5 |
| MD5.Oúnjẹ kìí wù mí láti jẹ | 1 | 2 | 3 | 4 | 5 |
| E1.Ó máa n sábà rẹ̀ mí | 1 | 2 | 3 | 4 | 5 |
| E2.Mo máa n ní láti dúró sinmi ní ọ̀pọ̀lọpọ ìgbà lójú ọjọ́ | 1 | 2 | 3 | 4 | 5 |
| E3.Ó rẹ̀ mí débi pé n kò leè ṣe àwọn ohun tí mo fẹ́ ẹ́ ṣe | 1 | 2 | 3 | 4 | 5 |

Irú òsùnwọ̀n wo ni o le fi lé àwọn nnkan wọ̀nyìi bí o bá fi wé àkókò tí o kò tíì ní àìsàn rọpá-rọsẹ̀ yìí? Fi ami ‘X’ si aaye kookan ti o ba idahun re mu lati so boya nnkan kookan burú jáì, burú n’íwọ̀nba, burú diẹ̀ tabi se deede pẹ̀lú kí o tó ní àìsàn rọpá-rọsẹ̀.

|  | Ó burú jáì ju kí n tó ní àìsàn rọpá- rọsẹ̀ lọ  1 | Ó burú n’íwọ̀nba ju kí n tó ní àìsàn rọpá-rọsẹ̀ lọ  2 | Ó burú die ju kí n tó ní àìsàn rọpá-rọsẹ̀ lọ    3 | Deede pẹ̀lú kí n tó ní àìsàn rọpá-rọsẹ̀  4 |
| --- | --- | --- | --- | --- |
| 1E. Okun Inú mi/Agbára mi láti ṣiṣẹ́ |  |  |  |  |
| 2FR. Ipa mi nínú ẹbí |  |  |  |  |
| 3L. Ọ̀rọ̀ sísọ mi |  |  |  |  |
| 4M. Ìrìn rírìn mi |  |  |  |  |
| 5MD. Ipò ọkàn mi/Múùdù mi |  |  |  |  |
| 6P. Ìrínisí mi |  |  |  |  |
| 7SC. Àmójútó ara mi |  |  |  |  |
| 8SR. Ojúṣe mi l’áwùjọ |  |  |  |  |
| 9T Àsàrò ọkàn mi/Èrò ọkàn mi |  |  |  |  |
| 10UE. Ìlò ọwọ́ àti apá mi |  |  |  |  |
| 11V. Ìran wíwò mi/ Ìríran mi |  |  |  |  |
| 12W. Iṣẹ́ ṣíṣe mi |  |  |  |  |
| 13 Àpapọ̀ òdiwọ̀n ìgbáyégbádùn  ajẹmọ́ ìlera mi |  |  |  |  |
