# Supplementary material for: Validity and reliability of a Nigerian-Yoruba version of the stroke-specific quality of life scale 2.0
Source: Health Qual Life Outcomes. 2017 Oct 19;15:205. doi: 10.1186/s12955-017-0775-9 (PMC5649048; doi:10.1186/s12955-017-0775-9)
Supplement: Supplementary file 2 — Yorùbá Version Of Whoqol-Bref. (DOCX 19 kb) [file 12955_2017_775_MOESM2_ESM.docx]

ADDITIONAL FILE 2 **YORÙBÁ VERSION OF WHOQOL-BREF**

**JÒWÓ KA ÌBÉÈRÈ KÒÒKAN, SE ÒDÍNWÒN ÌMÒLÁRA RE, KÍ Ó SÌ FÀÀLÀ SÌ ÌWÒN TÍ O BÁ MU FÚN ÌBÉÈRÈ KÒÒKAN**

|  |  | Burù púpò | Burú | Kò burú ko dará | Dará | Dará púpò |
| --- | --- | --- | --- | --- | --- | --- |
| 1(G1) | Báwo ni o se ma a òdiwòn igbé ayé ré? | 1 | 2 | 3 | 4 | 5 |

|  |  | Kò té mi lórùn rárá | Kò té mi lórùn | Kò té mi lórùn sùgbón kò burú rárá | O té mi lórùn | O té mi lórùn púpò |
| --- | --- | --- | --- | --- | --- | --- |
| 2(G4) | Báwo ni ìlera re se té o lórùn sí? | 1 | 2 | 3 | 4 | 5 |

**Àwọn ìbéèrè wònyí n bí** ó **ní** ò**diw**ò**n bí o ti se ní ìrírí àwon nnkan kan ní òsè méjì séyìn**

|  | Ti Ara | Rárá | Díè | N’íwòn | Ò pò | Ò pò gan-an |
| --- | --- | --- | --- | --- | --- | --- |
| 3(F1.4) | Ò tó báwo ti o rò pé irora ara n dí o lówó láti se ohun tí o ní láti se? | 1 | 2 | 3 | 4 | 5 |
| 4(F1.3) | Báwo ni o se nílò ìtójú étò ìlera ìgbàlódé si fún ìgbe ayé . | 1 | 2 | 3 | 4 | 5 |
| 5(F4.2) | Báwo ni o se n gbádùn ayé sí? | 1 | 2 | 3 | 4 | 5 |
| 6(F4.2) | Báwo ni o se rope ayé re ni ìtumò sí? | 1 | 2 | 3 | 4 | 5 |

|  |  | Rárá | Díè | Ó mo n’íwòn | Ò pò | Ò pò gan-an |
| --- | --- | --- | --- | --- | --- | --- |
| 7(F1.4) | Báwo ni o se e lè fokàn ba nnkan lo si? | 1 | 2 | 3 | 4 | 5 |
| 8(F16.7) | Báwo ni o se rò pé o ní ààbò sí lójoojumó. | 1 | 2 | 3 | 4 | 5 |
| 9(F22.1) | Báwo ni ìlera agbègbè re se rí? | 1 | 2 | 3 | 4 | 5 |

**Àwọn ìbéèrè wònyí n bí o nípa bí o se ni ìrírí tàbí bí o se lè se nnkan kan ní òsè méjì séyìn**

|  |  | Rárá | Díè | Ó mo n’íwòn | Ò pò | Ò pò gan-an |
| --- | --- | --- | --- | --- | --- | --- |
| 10(F2.1) | Njé o ní okun tí ó tó fún ojoojúmó? | 1 | 2 | 3 | 4 | 5 |
| 11(F7.1) | Báwo irisi re se te o lórùn sí?. | 1 | 2 | 3 | 4 | 5 |
| 12(F18.1) | Njé o ní owó tí ó tó fún iní re? | 1 | 2 | 3 | 4 | 5 |
| 13(F20.1) | Báwo ni ìròyìn/iwifúnni tì o nílò fún ayé re se wa l’áròówótó re si? | 1 | 2 | 3 | 4 | 5 |
| 14(F21.1) | Bàwo ni o se ní ànfààní làti se erè ìdárayá fún fàájì sí? | 1 | 2 | 3 | 4 | 5 |
| 15(F21.1) | Bàwo ni o se lè rìn káàkiri sí? | 1 | 2 | 3 | 4 | 5 |

|  |  | Kò té mi lórùn rárá | Kò té mi lórùn | Kò té mi lórùn sùgbón kò burú | O té mi lórùn | O té mi lórùn dáadáa |
| --- | --- | --- | --- | --- | --- | --- |
| 16(F3.3) | Báwo ni oorun re se té o lórùn sí? | 1 | 2 | 3 | 4 | 5 |
| 17(F10.4) | Báwo ni bi o se n se isé re lójoojúmó se té o lórùn sí? | 1 | 2 | 3 | 4 | 5 |
| 18(F12.4) | Báwo ni agbára/okun tí o ní láti se isé se té o lórùn sí? | 1 | 2 | 3 | 4 | 5 |
| 19(F6.3) | Báwo ni ara re se té o lórùn si? | 1 | 2 | 3 | 4 | 5 |
| 20(F13.3) | Báwo ni ìbásepò re pèlú ara àti òré se té o lórùn sí? | 1 | 2 | 3 | 4 | 5 |
| 21(F15.3) | Báwo ni ìgbé ayé re nípa íbálòpò loko-laya se té o lórùn sí? | 1 | 2 | 3 | 4 | 5 |

| 22(F14.4) | Báwo ni áwon átìléyìn tí o n ri gbà láti òdò àwon òré re se té o lórùn sí? | 1 | 2 | 3 | 4 | 5 |
| --- | --- | --- | --- | --- | --- | --- |
| 23(F17.3) | Bàwo ni bí ibi tí o n ri gbé se té o lòrùn sí? | 1 | 2 | 3 | 4 | 5 |
| 24(F19.3) | Báwo nu bi ètò ìlera tí ò n gbà se té o lórùn sí? | 1 | 2 | 3 | 4 | 5 |
| 25(F23.3) | Báwo ni irorun àti wo okò re tè o lórùn sí? | 1 | 2 | 3 | 4 | 5 |

**Ìbéèrè yìí n bí ó ìrírí re nípa nnkan kan ní òsè méjì séyìn.**

|  |  | Kò rí béè ri | Ó rí béè díèdíè | Ó rí béè léèkòòkan | Ó rí béè lóòrèkóòrè | Ó rí béè ni gbogbo ìgbà |
| --- | --- | --- | --- | --- | --- | --- |
| 26(F8.1) | Òtó bii ìgbà mélòó tí èròkerò bii ìbànújé, Ìgbókànsókè ati ìpòrúru okàn máa n wá sí o lókàn? | 1 | 2 | 3 | 4 | 5 |

**Njé o ní òròkọrò lórí àgbéyéwò yìí?**

………………………………………………………………………………………………………………………………………………………………………………………………………………………………………………………………………………………………………………………………………………………………

**O SÉ É FÚN ÌRÀNLÓWÓ RÈ.**
